# Everybody's Page

**Published:** 1892-03-26

**Authors:** 


### Everybody's Page.

A correspondent to the *North American Practitioner* recommends a local application of carbolic acid applied in the form of a four per cent. solution on a warm flannel for cases of inflammatory rheumatism. The best time for using the remedy is on retiring to rest at night.

According to a German physician, liquid paraffin when slightly warmed is a solvent of camphor which has none of the objections possessed by solutions hitherto generally employed. A solution of camphor when thus obtained is perfectly clear and limpid, and has the property of keeping good for a long time.

Not infrequently of late it has been stated from various quarters in Australia that certain plants are remedies for the bites of venomous snakes. Sufficient grounds for these statements do not, unfortunately, seem to exist, for Sir Joseph Fayrer, who knows something about this matter, assures us that no antidote is yet known for such bites. So far science has not advanced any farther than to teach us to cut out the affected portion of the flesh at once or to cauterise it.

Chloride of ethyl as an anæsthetic, the use of which has hitherto been confined chiefly to dentistry and as an external application for neuralgia, seems likely to take the place in general surgery of ether and cocaine which, especially the latter, have not fulfilled the promises held out for them. M. Monnet believes that in chloride of ethyl an anæsthetic has been found which is purely local in its action and absolutely non-poisonous.

It would seem far from improbable that ere long many drugs which are now taken internally will be administered by means of subcutaneous injections. That many drugs are more efficacious and beneficial in their action on the patient when so administered seems beyond question. The result of a long series of investigations has led Dr. Rosenthal to recommend certain preparations of iron for hypodermic injection, more especially in cases of persons suffering from dyspepsia associated with anæmia and of delicate neurasthenics.

Dr. Bonnejoy appears to be a more than ordinarily enthusiastic believer in vegetable diet, judging from his book on the subject recently published in Paris. Flesh-eaters will be pardoned if they hesitate to accept all the dicta of the author, who, in his eagerness to see all men vegetarians, dubs believers in the opposite cult with the highly-refined epithet of "corpse-eaters." Absolute vegetarianism may be the better of the two diets for those who lead a meditative and comparatively easy existence, but rice and beans which sustain a native of India will not, we believe, be found sufficient sustenance for those who are the victims of the restless activity and unceasing energy of modern life in the Western world. It has yet to be proved that a vegetarian diet affords men extra resistance to disease, and for that matter that meat diet is the origin of the majority of our diseases. That we might with advantage to our health practice more moderation in the use of meat is a point which can hardly be contested, but this hardly warrants the use of such epithets as the one we have quoted.

